# NF-κB, Mesenchymal Differentiation and Glioblastoma

**DOI:** 10.3390/cells7090125

**Published:** 2018-08-31

**Authors:** Bakhtiar Yamini

**Affiliations:** Section of Neurosurgery Department of Surgery, The University of Chicago, Chicago, IL 60637, USA; byamini@surgery.bsd.uchicago.edu; Tel.: +1-773-834-3288

**Keywords:** NF-κB, GBM, glioma, mesenchymal, proneural, EMT, microenvironment

## Abstract

Although glioblastoma (GBM) has always been recognized as a heterogeneous tumor, the advent of largescale molecular analysis has enabled robust categorization of this malignancy into several specific subgroups. Among the subtypes designated by expression profiling, mesenchymal tumors have been associated with an inflammatory microenvironment, increased angiogenesis, and resistance to therapy. Nuclear factor-κB (NF-κB) is a ubiquitous transcription factor that plays a prominent role in mediating many of the central features associated with mesenchymal differentiation. This review summarizes the mechanisms by which NF-κB proteins and their co-regulating partners induce the transcriptional network that underlies the mesenchymal phenotype. Moreover, both the intrinsic changes within mesenchymal GBM cells and the microenvironmental factors that modify the overall NF-κB response are detailed.

## 1. Introduction

Epithelial-mesenchymal transition (EMT) was first described in the setting of embryonic development as an essential process required for multiple stages of organ and tissue differentiation [1]. EMT involves the loss of processes that promote cellular polarity and cell-cell contact and the gain of mesenchymal characteristics, including the ability to migrate and invade [2]. EMT also plays a central role in the response to injury and wound healing. In addition to these, mesenchymal differentiation is a prominent feature of cancer, acting to promote tumor growth, migration, and metastasis [3]. While cancer-associated EMT has primarily been studied in the setting of carcinoma, mesenchymal differentiation is also seen in other cancer types, including glioblastoma (GBM) [4].

Standard therapy for GBM involves maximal surgical resection followed by radiation therapy (RT) and alkylating chemotherapy with temozolomide (TMZ). Survival for patients with GBM remains at little over one year with only incremental improvement achieved with each novel agent [5,6]. Despite this dismal overall prognosis, subgroups of patients exist that have significantly better responses to therapy and outcome [7,8,9]. In an attempt to separate these tumors into different categories, Phillips et al. performed gene expression profiling on a series of GBMs, and grade III gliomas, and identified three distinct expressional subtypes: mesenchymal (MES), proliferative (PRO) and proneural (PN) [4]. Subsequently, the Cancer Genome Atlas (TCGA) group used unsupervised clustering of global gene expression from 200 GBMs and identified four clusters: PN, Neural (N), Classical (CL) and MES [10]. Notably, in these and subsequent analyses, PN and MES tumors repeatedly stood out as being the most consistent subtypes [11]. The importance of GBM subgrouping is not only in its diagnostic ability, but it also provides information on prognosis and potentially, response to specific therapy. In this regard, the PN subtype, or reduced MES component, has been repeatedly associated with better response to therapy and improved survival [12,13,14,15].

While these initial molecular studies examined bulk GBM specimens, it was subsequently demonstrated that even within a single tumor different subtypes coexist in spatially segregated areas [16]. Notably, even at the single cell level there is heterogeneity of expressional subtype [14]. A prominent feature of these expression programs is that they are plastic [15]. Specifically, like EMT, GBM cells can undergo proneural-mesenchymal-transition (PMT), either spontaneously [12,17] or in response to DNA damaging therapy [4,18,19]. Moreover, PMT upon disease recurrence has been implicated in treatment resistance and GBM relapse [4,13,17].

As with carcinoma, mesenchymal differentiation in GBM is identified by elevated expression of proteins such as vimentin, CD44, and matrix metalloproteinases (MMPs) and decreased expression of epithelial markers like E-cadherin [20]. Moreover, mesenchymal differentiation is orchestrated by a series of transcription factors, including SNAI1 (SNAIL), TWIST1 and zinc finger E-box-binding homeobox-1 (ZEB-1) and ZEB-2. In addition, in GBM, unbiased interrogation of gene regulation in mesenchymal tumors demonstrated that they are regulated by specific master transcription factors, including transcriptional coactivator with PDZ-binding motif (TAZ), C/EBPβ and STAT3 [21,22]. Closely intertwined in this complex mesenchymal transcriptional network reside the nuclear factor-κB (NF-κB) family of proteins.

## 2. NF-κB

NF-κB is a multi-subunit transcription factor made up of five primary proteins: p50 (NF-κB1, p105), p52 (NF-κB2, p100), p65 (relA), relB, and crel [23]. These subunits mediate their cellular effects by binding to DNA as dimers. While all subunits contain an N-terminal Rel homology domain (RHD) necessary for DNA binding and subunit dimerization, only p65, relB, and c-Rel contain a C-terminal transactivation domain (TAD) [24]. In general, NF-κB dimers are maintained in the cytoplasm bound to inhibitor-κB (IκB) proteins. Activation of NF-κB occurs by multiple interrelated pathways that converge on the cytoplasmic IκB kinase (IKK) complex made up of two catalytic subunits, IKKα and IKKβ, and a non-catalytic, regulatory subunit, IKKγ (NEMO) [23]. Phosphorylation of IκB proteins results in their degradation leading to NF-κB nuclear translocation. While nuclear translocation is the primary method of NF-κB regulation, the overall NF-κB response is determined by the cooperative action of multiple promoter-specific factors, such as the composition of the NF-κB dimer, the specific post-translational modification (PTM) of each subunit and the identity of the co-regulating factor present at each promoter [25,26]. In addition, the specific sequence of the *cis*-acting κB-site also plays an important role in regulating NF-κB activity and gene expression [27,28].

The primary NF-κB dimer found in resting GBM cells is comprised of p50 and p65 [29,30]. p50 is constitutively produced in a co-translational manner from its parental protein, p105 [31,32]. As p50 lacks a TAD, it functions in an inhibitory capacity unless it is either dimerized with a TAD-containing subunit, such as p65, or associated with a transactivating coregulator. Although p65 is generally retained in the cytoplasm at rest, in malignant cells there is an elevated level of continuous cytokine and oncogene stimulation, resulting in increased IKK activity and nuclear p65 translocation. Given the critical role of p65 in promoting NF-κB transcriptional activity, the majority of work examining NF-κB in GBM has focused on this subunit.

While nuclear translocation and DNA binding are necessary for p65 to promote its effects, site-specific phosphorylation is also associated with increased NF-κB activity. There are over ten p65 phosphorylation sites [26], among which Serines 276 and 536 have been best characterized. Although S276 phosphorylation is associated with increased NF-κB activity in multiple settings [33,34], this site is not routinely used as an indicator of NF-κB activation in GBM, possibly because of the discrepancy associated with the most commonly used anti-phospho-S276-p65 antibody [35]. However, phosphorylation of S536, also induced by a variety of stimuli and kinases [36], is routinely used as an indicator of increased NF-κB activity in the setting of GBM. While the importance of p65 to global NF-κB cellular activity and the ease of examining activated phospho-p65 have made it the primary method for analyzing NF-κB in GBM tissue [13,37], other subunits, such as p52 or relB, that are not as easily examined in vivo as phospho-p65, also play an important role in overall GBM pathobiology [10,38,39].

**Figure 1:**
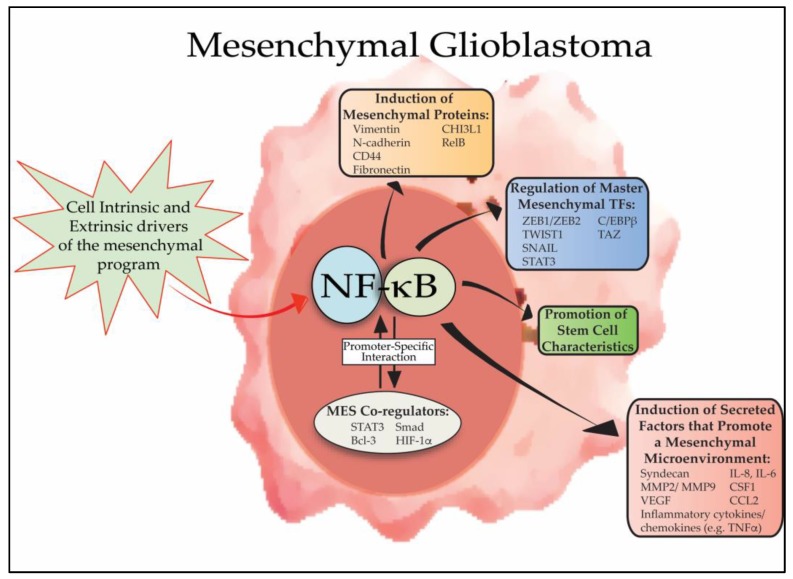
Overview of the role of NF-κB in GBM mesenchymal differentiation. NF-κB is activated in mesenchymal GBM cells by a series of cell intrinsic and extrinsic signals (e.g., genetic alterations and microenvironmental cytokines, respectively) that promote mesenchymal differentiation. Subsequently, nuclear NF-κB promotes mesenchymal differentiation by inducing the expression of master mesenchymal transcription factors, including, *STAT3*, *CEBPB* and *TAZ,* and by directly inducing expression of mesenchymal proteins such as CD44, vimentin, N-cadherin. In addition, NF-κB promotes mesenchymal changes in the tumor microenvironment by regulating the composition of secreted cytokines, ECM proteins and other enzymes to promote invasion, angiogenesis and resistance to therapy. Active NF-κB promotes mesenchymal differentiation in conjunction with other transcription factors and co-regulators, such as STAT3, Bcl-3 and HIF-1α.

From a general perspective, NF-κB sits at the junction of upstream inducers and downstream mediators of the EMT response. In this regard, NF-κB is activated by a variety of cell-intrinsic and microenvironmental factors that promote mesenchymal differentiation. Subsequently, activated NF-κB functions to regulate a network of transcription factors and other proteins that mediate the overall mesenchymal program (Figure 1). In the following section, the specific mechanisms by which the NF-κB system acts in this capacity will be highlighted.

## 3. NF-κB and Regulation of Mesenchymal Factors

Mesenchymal transition is classically regulated by specific transcription factors, including SNAIL, ZEB1 and TWIST1 [3]. SNAIL proteins repress epithelial genes by binding to E-box sequences in their promoter regions [40]. In Drosophila, the NF-κB homologue, Dorsal, induces snail expression [41], while in human cells, p65 binds to the proximal SNAIL promoter to induce its transcriptional activity [42]. NF-κB was also shown to mediate the increase in SNAIL induced by inhibition of glycogen synthase kinase-3 (GSK-3) [43]. Consistent with the role of GSK-3 in this response, constitutively active insulin-like growth factor receptor (IGF-1R) activates AKT, a negative regulator of GSK-3, and NF-κB resulting in increased *SNAIL* mRNA expression [44]. In addition, SNAIL was shown to promote an increase in cancer cell invasion and migration in response to the inflammatory cytokine TNFα [45]. Interestingly, this latter pathway was shown to be mediated by NF-κB-induced stabilization of SNAIL protein.

Another critical mesenchymal transcription factor induced by NF-κB is the basic helix-loop-helix (bHLH) factor, TWIST1. bHLH transcription factors bind E-boxes and play a critical role in downregulating epithelial genes, while also inducing mesenchymal factors [46]. In drosophila as with SNAIL, TWIST is directly induced by Dorsal [47,48]. Moreover, in mice lacking IKKα, a decrease in the expression of twist protein is seen [49]. In mouse embryonic fibroblasts, TNFα induces twist1 by a mechanism that requires p65 [50] and in human mesenchymal lung cancer cells, expression of the IκBα super-repressor (IκBα-SR), which specifically inhibits canonical NF-κB signaling, blocks TNFα-induced TWIST1 expression [51]. Notably, inflammation-induced mesenchymal differentiation was reported to be mediated by p65-induced expression of TWIST1, a response that promotes metastasis and is associated with poor prognosis in breast carcinoma [52].

NF-κB also induces the expression of the ZEB family of transcription factors, comprised of ZEB1 and ZEB2. Specifically, both factors contain κB-sites in their promoters, and p65 was reported to induce their mRNA expression and the activity of a luciferase reporter bearing the *ZEB1* κB-site [53,54]. Moreover, NF-κB was shown to bind the *ZEB1* promoter and induce expression from a *ZEB1* promoter reporter in GBM stem-like cells (GSCs) to promote migration [55]. Similarly, NF-κB was shown to increase ZEB1 protein expression in pancreatic cancer cells [56].

In addition to factors classically described as regulators of mesenchymal differentiation, unbiased systematic analyses in GBM identified other proteins associated with mesenchymal differentiation, including STAT3, C/EBPβ and TAZ [21,22]. Interestingly, in patient-derived GSCs, the mRNA expression of *STAT3*, *CEBPB* and *TAZ* was blocked by IκBα-SR, suggesting that in GBM, master mesenchymal transcription factors are regulated by NF-κB [13]. The close link between NF-κB and STAT3 in cancer has been previously reviewed [57]. These two transcription factors act together to induce angiogenesis and inflammatory cell infiltration, processes that are central to mesenchymal differentiation. They also often act in concert to promote cytokine expression [58,59,60]. In GBM, STAT3 was shown to act with p65 to upregulate the Notch pathway and promote glioma stem cell characteristics [61]. STAT3 has also been reported to induce the formation of p52 [62], an NF-κB subunit recently shown to be required for mesenchymal gene expression in GBM [63].

While the above findings indicate that NF-κB regulates and interacts with many of the master transcription factors linked to mesenchymal differentiation in GBM, downstream of these master regulators a battery of structural and secreted proteins mediate the mesenchymal phenotype. In general, mesenchymal transition involves loss of the epithelial marker, E-cadherin, and concomitant increase in mesenchymal factors, N-cadherin and vimentin (*VIM*) [3]. In addition to modulating the expression of these factors via intermediate transcription factors, NF-κB can also directly promote expression of several mesenchymal proteins. In this regard, the N-cadherin (*CDH2*) promoter has been shown to contain an NF-κB consensus site [64], and *VIM* is a well-characterized NF-κB target gene [65], induced by both p65 and relB to promote mesenchymal differentiation [53,66]. Finally, NF-κB also directly regulates several of the factors identified as being specifically upregulated in mesenchymal GBM, including *CHI3LI*, *CD44* and *RELB* [10,67,68,69].

## 4. NF-κB, Mesenchymal Differentiation and Stem Cells

One of the fundamental features of EMT is acquisition of a more pluripotent phenotype that is associated with stem-like cells [70]. In this regard, cancer cells that undergo mesenchymal differentiation upregulate factors associated with stem cells and MES GBM are linked to elevated expression of stem cell marker genes [4,71]. NF-κB is a transcription factor closely linked to promoting the maintenance and propagation of stem-like cancer cells [72] (Figure 1). In breast cancer, both the canonical and non-canonical pathways have been reported to be involved in promoting stem cell self-renewal by a mechanism involving EMT [73]. Similarly, in prostate cancer, cells with stem-like characteristics demonstrated increased NF-κB activity related to a decrease in *NFKBIA* transcription and IκBα levels [74]. In GBM, NF-κB signaling has also been implicated in GSC propagation. On the one hand, in response to epithelial V-like antigen 1 (Eva1), the non-canonical NF-κB activator NF-κB inducing kinase (NIK) was shown to promote GBM tumorigenic and stem cell properties by increasing relB levels [75]. On the other hand, constitutive (nuclear) p65 was shown to interact with STAT3 in GBM cells to promote GSC growth [61].

Another signaling response intimately associated with stem cell growth in GBM and cancer, in general, is the WNT/β-catenin pathway [76]. WNT5A was found to promote GSC differentiation and tumor recurrence [77]. In addition, inhibition of the WNT pathway in GSCs resulted in a decrease in mesenchymal differentiation and reversal of stem-like properties [78]. Elevated NF-κB signaling in a model of intestinal cancer was shown to enhance Wnt activation and induce dedifferentiation of non-stem cells into tumor-initiating cells, further supporting the link between NF-κB and stem cell properties [79].

Transforming growth factor-β (TGFβ) is a central factor involved in the self-renewal and maintenance of GSCs and is recognized as a potent inducer of EMT in cancer [40,80]. In GBM, TGFβ has been shown to promote a stem cell phenotype in patient-derived neurospheres by inducing expression of leukemia inhibitory factor (LIF) [81], Sox2 and Sox4 [82]. Interestingly, in the original transcriptional profiling of GBM, *LIF* was shown to be a primary MES signature gene [4]. Cross-talk between the TGFβ and NF-κB pathways occurs via multiple downstream signaling mechanisms [83] and in general, TGFβ has been reported to activate NF-κB. Pathways induced by TGFβ activate TGF-β-activated kinase 1 (TAK1), a kinase involved in mediating innate immune signaling [84], which phosphorylates and activates p65 and IKKα [85]. This latter pathway has been reported to promote EMT [86]. While TAK1 activation demonstrates a Smad-independent pathway for activation of NF-κB, TGFβ also uses Smad-dependent responses to modulate NF-κB signaling [83,86,87].

While the above pathways illustrate the importance of NF-κB in promoting stem cell characteristics in GBM, another aspect of NF-κB signaling that is specific to GSCs is its role in promoting PMT. This response was initially reported in relation to NF-κB activation in GSCs by TNFα in the surrounding microenvironment [13]. This study highlighted the heterogeneity of GSCs in GBM and the propensity for mesenchymal differentiation under specific conditions. A follow-up study by the same group subsequently identified the serine/threonine kinase, mixed lineage kinase 4 (MLK4), as being an intrinsic factor, important specifically in the stem cell pool of mesenchymal GBM [88].

## 5. NF-κB, Mesenchymal Differentiation and GBM Genetic Modification

**Figure 2:**
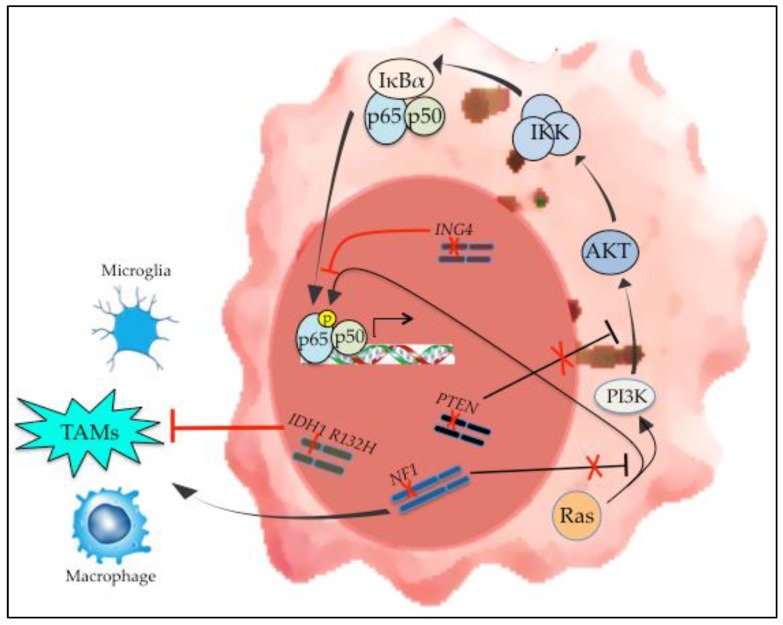
Genetic alterations in mesenchymal GBM and their effect on NF-κB. Loss of NF1 and PTEN are characteristic findings in mesenchymal GBM. In addition, IDH1 mutation is the defining alteration of IDH-mutant GBMs that cluster transcriptionally with PN tumors. The effect of these genetic changes on NF-κB activation is demonstrated, as is the effect of these alterations on the tumor microenvironment.

Mesenchymal differentiation in GBM is associated with specific genetic alterations (Figure 2). Verhaak and TCGA investigators found that hemizygous deletion or mutation of neurofibromin 1 (NF1) was the primary modification seen in MES tumors [10]. Moreover, in this study, even the expression level of NF1 was significantly lower in MES GBM. Consistent with this observation, in mouse models of GBM, loss of NF1 promotes mesenchymal differentiation [17,89]. Interestingly, in the study by Ozawa et al., it was suggested that master MES regulators such as STAT3 and C/EBPβ, that are regulated by NF-κB [13], are also downstream of NF1 [17]. Functionally, NF1 blocks Ras signaling [90]. Consequently, loss of NF1 results in increased NF-κB activity due to dis-inhibition of the Ras pathway [23,91,92]. In addition, co-mutation of NF1 and PTEN was most frequently seen in the MES subtype [10]. Loss of PTEN also activates NF-κB by promoting Akt signaling [23]. Finally, while genetic alterations of NF-κB subunits are rare in GBM, loss of the gene encoding IκBα, NFKBIA, has been linked to GBM [93]. However, alterations of NFKBIA have not been examined in relation to expression subtype and the mRNA expression of NFKBIA is actually elevated in MES tumors (GlioVis data portal for visualization and analysis of brain tumor expression datasets) [94].

While loss of tumor suppressors in neoplastic cells can promote cell-intrinsic NF-κB activation and mesenchymal differentiation, genetic alterations in these cells can also activate NF-κB indirectly. Mutation of the isocitrate dehydrogenase (IDH) 1 or 2 genes is a defining, early feature of a specific category of glioma [95,96]. These IDH-mutant tumors have global hypermethylation consistent with a CpG island methylator phenotype (CIMP) and have significantly better survival than IDH-wildtype tumors [8]. Notably, IDH-mutant tumors cluster transcriptionally with PN GBM [8]. It was recently shown that IDH-mutant gliomas have reduced levels of TAMs compared to IDH-wildtype [97]. Given the link between TAM infiltration, NF-κB activation and mesenchymal differentiation (see below) [13,98], the lower concentration of TAMs and cytokines in IDH-mutant tumors [97] likely results in reduced NF-κB activity and a more PN phenotype. This response demonstrates how genetic changes in GBM cells can influence the NF-κB-dependent transcriptional profile by modulating cell-extrinsic factors.

## 6. NF-κB and the Mesenchymal Microenvironment

**Figure 3:**
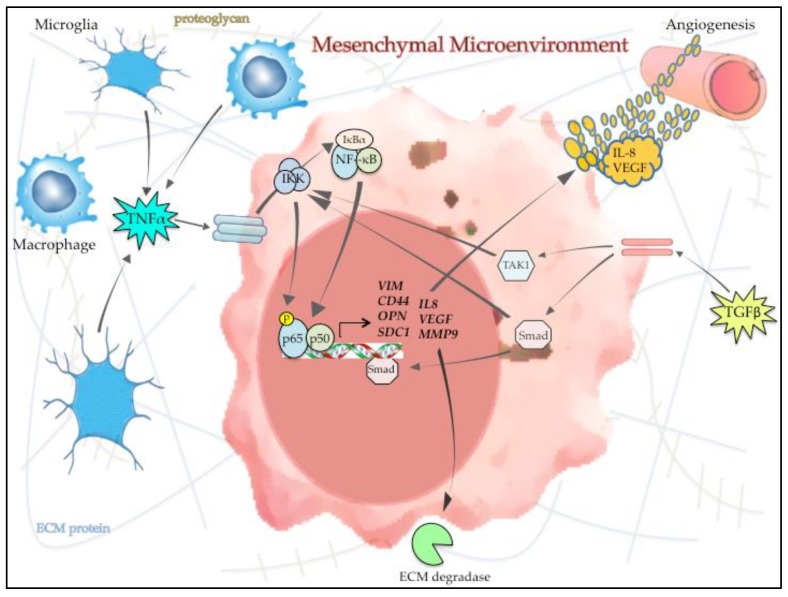
NF-κB and the microenvironment in mesenchymal GBM. Cytokines and other signaling molecules in the microenvironment (e.g., TNFα and TGFβ) are released by infiltrating tumor associated macrophages/microglia (TAMs) and other supporting cells and induce NF-κB activity in GBM cells. Activated NF-κB subsequently promotes mesenchymal change by inducing the expression and secretion of angiogenic factors (e.g., IL-8 and VEGF), ECM proteins/proteoglycans and ECM degrading enzymes, such as MMP9. This illustration demonstrates how the interaction between GBM cells and the surrounding microenvironment contributes to promoting the overall mesenchymal phenotype in GBM focusing on the role of NF-κB in this reciprocal process.

In the native setting, GBM is comprised of a network of neoplastic, vascular and inflammatory cells that are maintained in a complex extracellular matrix (ECM) [99]. While neoplastic cells harbor the genetic changes that underlie the pathology of each tumor, the overall malignant phenotype is intimately linked to and regulated by the surrounding microenvironment [100,101,102]. Specifically, the expression profile and molecular subtype of each GBM is regulated by the composition of the microenvironment. In a seminal study, it was reported that infiltrating tumor-associated macrophages and microglia (TAMs) promote mesenchymal differentiation by releasing cytokines into the surrounding microenvironment that induce p65 phosphorylation and NF-κB activation in the GBM cells [13]. Other groups corroborated the link between mesenchymal differentiation and infiltrating TAMs in GBM [15,98,103]. Notably, the entire extracellular milieu associated with the mesenchymal subtype is enriched with cytokines and inflammatory markers linked to elevated NF-κB activity within GBM cells [104] (Figure 3).

In addition to TAMs, another important feature of the mesenchymal microenvironment is increased angiogenesis [4,105] (Figure 3). Interleukin 8 (IL-8) is a chemokine with extensive pro-angiogenic properties and the *IL8* promoter contains an NF-κB binding site [106]. Activation of p65 with loss of the tumor suppressor, *ING4*, in GBM cells has been shown to induce secretion of IL-8 resulting in increased angiogenesis [107]. Interestingly, *IL8* is one of several chemokines upregulated not only in GBM cells but also in clinical mesenchymal GBM samples [104]. Another central angiogenic protein, vascular endothelial growth factor (VEGF), also contains an NF-κB consensus site within its proximal promoter [108]. Inhibition of p65 using a specific anti-p65 intrabody was shown to decrease angiogenesis and VEGF by blocking NF-κB transcriptional activity [109]. Similarly, expression of a non-degradable IκBα mutant decreased VEGF expression in GBM xenografts in vivo [110]. Finally, the link between NF-κB, angiogenesis and GBM mesenchymal differentiation is supported by the observation that cells with stem-like properties that are an important component of mesenchymal tumors occupy a specific perivascular niche in close association with vascular endothelial cells [102]. Notably, both CD44 and its ligand osteopontin (OPN) that are both NF-κB-regulated [69,111] and linked to stemness, were shown to be expressed primarily in this perivascular niche in GBM [112].

The ECM is comprised of a series of proteins and proteoglycan molecules that form a lattice that engulfs neoplastic and supporting cells. ECM proteins maintain the structure of the tumor mass and enable cell-cell signaling. Tumor growth and invasion involves remodeling of the ECM [40]. The proteins of the ECM, including cadherins, vimentin, fibronectin and other signaling molecules such as TGFβ are secreted by the surrounding tumor and supporting cells. As noted earlier, many of these proteins are regulated by the NF-κB pathway [113]. The proteoglycan, syndecan (SDC1) is regulated by NF-κB [114] and was found to be increased in mesenchymal GBM [115]. In addition to matrix proteins, enzymes such as the matrix metalloproteases (MMPs) are also upregulated with mesenchymal differentiation. It is well established that several MMPs have functional NF-κB binding sites in their promoters [116]. NF-κB was shown to induce MMP-2 and MMP-9 activity leading to increased fibronectin processing and GBM cell invasion [117] (Figure 3). In addition, in response to Bmi-1 (B cell-specific Moloney murine leukemia virus integration site 1) stimulation, MMP-9 was induced in an NF-κB-dependent manner to promote GBM invasion [118].

In the heterogeneous microenvironment of GBM, regions of hypoxia are common and are primarily associated with necrosis and pseudopalisading tumor cells. Although hypoxic regions are often highly vascular, the tumor vessels in these areas are tortuous and thrombosed, further increasing the propensity for hypoxia [119]. Hypoxia in GBM is associated with an increase in stem cell proliferation and tumor aggressiveness [120], and has been linked to mesenchymal differentiation [121]. In response to hypoxia, NF-κB is activated by a mechanism involving the IKK complex and TAK1 [122,123]. In clinical GBM, a link between hypoxia and activation of NF-κB-dependent inflammatory genes has also been reported [124]. The primary transcription factor associated with hypoxia is hypoxia-inducible factor (HIF), a family of proteins that are stabilized in the presence of low oxygen tension. While HIF proteins are primarily controlled at the post-translational level [125], NF-κB has been shown to regulate HIF-1α expression by modulating *HIF1A* promoter activity [126]. Moreover, loss of IKKβ was reported to lead to a defect in the induction of HIF-1α target genes [127]. In addition, HIF-1α has been shown to promote p65 activity [128] and to induce NF-κB-dependent secretion of inflammatory cytokines and chemokines [129]. Together, these findings illustrate the extensive crosstalk between the HIF and NF-κB pathways and underline their co-regulatory role in promoting mesenchymal differentiation in association with tissue hypoxia [50,130,131,132].

## 7. DNA Damaging Therapy, NF-κB and Mesenchymal Transition

**Figure 4:**
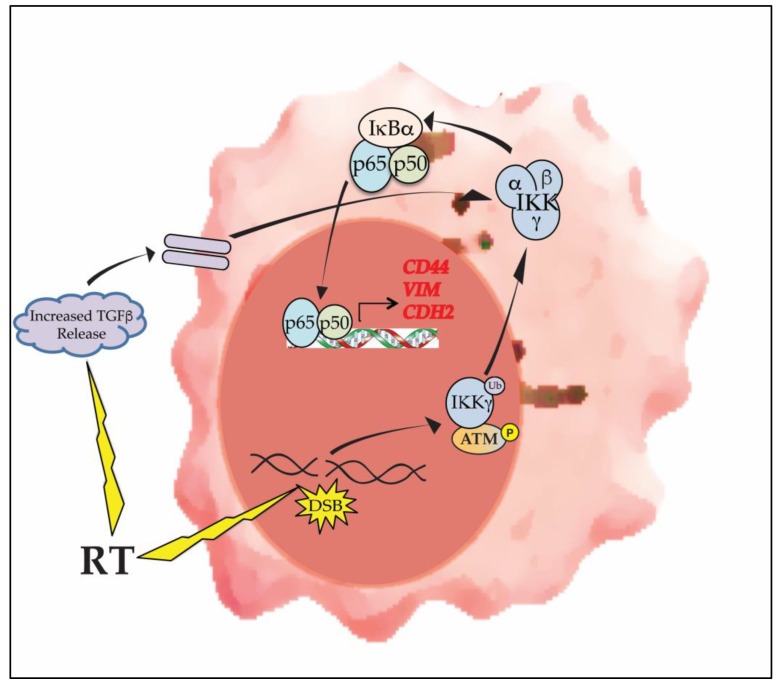
RT promotes mesenchymal change in GBM via activation of NF-κB. DNA damaging therapies such as RT induce NF-κB activation by forming DNA DSBs. This ‘atypical’ activation pathway involves phosphorylated ATM and mono-ubiquitinated IKKγ (NEMO). RT also induces the release of microenvironmental factors such as TGFβ that cooperate with NF-κB in promoting expression of mesenchymal proteins.

The original classification of GBM into expressional subgroups found that a significant number of PN tumors recurred with a MES expression profile, suggesting that GBM undergoes PMT upon recurrence [4]. Given that recurrent tumors have previously been treated with DNA-damaging therapeutics such as RT, mesenchymal transition may be related to the survival and growth of a population of tumor cells with a mesenchymal profile, as has been reported for other cancers [133]. On the other hand, RT has been shown to directly induce mesenchymal gene expression (Figure 4). In one study using patient-derived GSCs, RT was shown to induce the expression of mesenchymal factors and promote mesenchymal differentiation in PN GSCs [18]. In another study, RT was found to induce expression of mesenchymal transcription factors, including SNAIL and TWIST, a finding also seen in recurrent tumors compared to matched primary tumors [134]. Similarly, in a genetically engineered mouse model of GBM, RT was shown to induce mesenchymal gene expression as early as 6 hours after treatment resulting in PMT [19]. Although no study has directly demonstrated that such damage-induced PMT is NF-κB dependent, RT induces NF-κB with a similar time course as mesenchymal gene expression [135]. Activation of NF-κB by RT occurs following formation of DNA double strand breaks (DSBs) via a well-studied nuclear to cytoplasmic response involving ataxia telangiectasia mutated (ATM) and IKKγ [136]. In a mouse model of GBM, RT-induced PMT involved upregulation of several NF-κB-dependent mesenchymal factors [137]. Moreover, a recent examination of GSCs treated with RT identified NF-κB as one of the most enriched transcription factors [138]. These findings suggest that therapy-induced PMT is not solely due to survival of populations of *a-priori* resistant cells, but in fact the result of a shift in the overall expressional program.

In addition to directly activating NF-κB via formation of DSBs, RT also promotes a mesenchymal program by inducing an increase in microenvironmental TGFβ [134,139]. As noted earlier, TGFβ cooperates with NF-κB via Smad-dependent and -independent pathways to induce mesenchymal master regulators.

NF-κB-mediated mesenchymal differentiation in GBM is not only induced by DNA damaging therapy but also leads to resistance to treatment, a phenomenon seen in both experimental studies and clinical samples [13,15]. Mesenchymal differentiation also leads to resistance to other therapeutics, including anti-angiogenic agents [12,140]. While mesenchymal differentiation and resistance to RT involves TNFα-induced activation of NF-κB [13], this response is blocked by the G-protein coupled receptor (GPCR), GPR56, a protein that inhibits NF-κB activation by acting on the IKK complex [141].

## 8. Mesenchymal Differentiation and Bcl-3

B cell CLL/lymphoma 3 (Bcl-3) is one of the best characterized NF-κB co-regulators [142]. Originally identified as a candidate oncoprotein in chronic leukemia patients [143], Bcl-3 is an atypical IκB protein that regulates NF-κB activity primarily in conjunction with p50- and p52-containing dimers [144,145]. An initial link between Bcl-3 and EMT was suggested when it was shown that Bcl-3 is recruited to the N-cadherin promoter to activate transcription [146]. Subsequently, Bcl-3 was found to promote epidermal growth factor (EGF)-induced EMT in cervical cancer cells [147]. In addition, in mouse mammary tumors, Bcl-3 was shown to promote motility and metastasis without altering the expression of cell adhesion factors such as E- or N-cadherin [148].

We recently identified Bcl-3 as a factor that promotes resistance to alkylating chemotherapy in GBM [63]. Mechanistically, we found that Bcl-3 promotes mesenchymal differentiation in patient-derived GSCs by inducing promoter-specific NF-κB dimer exchange. Specifically, elevated Bcl-3 was associated with increased nuclear p65 translocation and replacement of p50 by p52 at the κB-sites of mesenchymal factors like *CD44*, *CCL2* and *LIF*. While p65 phosphorylation, NF-κB activation and CD44 expression were previously shown to occur in GSCs in response to TNFα released by infiltrating TAMs [13], we found that high Bcl-3 augments these responses to further promote mesenchymal change. Consistent with this observation, in clinical GBM samples from TCGA, *BCL3* expression level correlated strongly with the expression of all the mesenchymal signature genes as designated by Phillips et al. [4]. In addition, we found that *BCL3* expression level was regulated by copy number alteration of 19q13, the chromosomal band where the *BCL3* gene is located. Given the critical role of Bcl-3 in regulating NF-κB signaling, identification of Bcl-3 as a factor that promotes PMT further expands the role of NF-κB in modulating GBM biology.

## 9. Concluding Remarks

The expression profile of GBM is influenced both by genetic alterations in the neoplastic cells and as a result of alterations in the surrounding microenvironment. While the genetic changes in a tumor are relatively fixed, the downstream transcriptional patterns are highly variable. Importantly, these changes in gene expression, which occur both spontaneously and in response to DNA damaging therapy, underlie the overall malignant phenotype of each tumor. Mesenchymal differentiation, or PMT, is a central phenomenon underlying the pathobiology of GBM. NF-κB is a ubiquitous transcription factor that regulates the response to a diverse range of stimuli. While NF-κB has most often been considered a stimulus-induced factor, even in resting cells there is significant basal NF-κB activity [31,32]. In this regard, the NF-κB pathway is ideally positioned to integrate the signals that are induced within GBM cells with the stimuli that arise from the surrounding microenvironment. While the NF-κB-dependent response can be quite diverse, in general NF-κB signaling promotes mesenchymal differentiation.

Given that NF-κB primarily induces an aggressive phenotype, significant effort has been placed at incorporating NF-κB inhibition into the treatment of GBM; however, to date there has been no clear success. Notably, the diverse and subunit-specific nature of the NF-κB response in cancer [149,150,151] suggests that targeting NF-κB proteins, or the IKK complex, can have unpredictable results. A potentially more fruitful approach to target the NF-κB pathway is to identify downstream NF-κB-dependent factors that promote deleterious effects. Using such a strategy, we identified carbonic anhydrase II (CAII) as a Bcl-3-dependent factor that inhibits the efficacy of TMZ [63]. We subsequently demonstrated that the CAII inhibitor, acetazolamide, significantly improves the efficacy of TMZ, specifically in tumors with high Bcl-3 expression that have mesenchymal features.

In summary, the above data demonstrate the complex role of the NF-κB response in promoting mesenchymal differentiation in GBM. In future studies, it will be important to further dissect these pathways, focusing on specific subgroups of patients, in an attempt to improve the overall management of this heterogeneous disease.
